# Screening for prostate cancer: a study on the free and total prostate specific antigen

**DOI:** 10.15190/d.2021.18

**Published:** 2021-12-31

**Authors:** Ridvana Mediu, Ariol Rama, Naim Mediu

**Affiliations:** ^1^Faculty of Applied Science, Medical Science Department, University College LOGOS, Tirana, Albania; ^2^General surgeon. Surgery Service, Regional Hospital, Durres, Albania

**Keywords:** Total PSA, Free PSA, tumor marker, prostate cancer.

## Abstract

Background: A variety of biomarkers have been developed to monitor growth of cancerous diseases and to detect them at an early stage. Prostate-specific antigen (PSA) is a valuable prostate cancer biomarker that is now widely used for population screening, diagnosis, and monitoring of patients with prostate cancer. Other factors than prostate cancer can cause elevation of PSA levels therefore, free prostate specific antigen measurements in serum have been proposed in order to improve the specificity of laboratory identification of prostate cancer. Aim: The aim of our study was to evaluate the diagnostic significance of both total PSA and Free PSA in discriminating prostate cancer from other prostate diseases. Materials and methods: Our study group consisted of 1201 males admitted at outpatient clinic aged between 35 and 84 years old (mean age 63 years). All laboratory measurements were performed on serum samples. The data were statistically analyzed by using descriptive statistics for Windows. Results: The mean total PSA concentration evaluated among 1038 patients was 16.17 ng/mL whereas only Free PSA concentration was evaluated in 163 serum samples and resulted in a mean value of 2.67 ng/ml. In order to calculate the correlation between total and free PSA, data among 69 /1038 patients were further analyzed through statistical program software package for data analysis. Conclusions: Measuring serum free PSA concentrations along with PSA concentrations may provide higher accuracy for detecting prostate cancer and might eliminate unnecessary biopsies in the men with PSA of more than 4.0 ng/mL

## INTRODUCTION

Prostate cancer occupies second place in mortality caused by malignant diseases among men. According the data from WCRF (https://www.wcrf.org/), there were 1.3 million new cases in 2018. The number of deaths accounts 20.7 in 100.000 men for year. Prostate cancer is diagnosed annually in more than 180.000 new cases worldwide, with the greatest incidence in the US and Northern Europe. The mortality due to prostate cancer constitutes 4.4% of all cancer related death^1^. According to the latest WHO data published in 2020 Prostate Cancer Deaths in Albania reached 203 or 0.83% of total deaths^2^.Prostate-specific antigen (PSA) is a glycoprotein that is produced by the prostate gland, the lining of the urethra, and the bulb urethral gland. Normally, very little PSA is secreted in the blood. Increases in glandular size and tissue damage caused by benign prostatic hypertrophy, prostatitis, or prostate cancer may increase circulating PSA levels^3,4^. PSA exists in serum in multiple forms: complexes to alpha-1-anti-chymotrypsin (PSA-ACT complex) 70-90%, unbound (free PSA) 10-30%, which is the portion of PSA not bound to serum proteins and enveloped by alpha-2-macroglobulin (not detected by immunoassays). The portion of PSA that is complexes with α_2_-macroglobulin can be measured only if the complex is cleaved and the PSA epitopes become accessible^5^. PSA is commonly referred to as a “biomarker” or “tumor marker”, because it provides information about the biological state of prostate cancer disease^6^. Early diagnosis and management of prostate cancer has been revolutionized, and much has been learned about the strengths and weaknesses of these PSA assays. PSA testing not only helps identify men in whom a prostate biopsy would be appropriate but also assists in assessing the response to therapy, determining tumor progression, and, in its most controversial role, screening for prostate cancer^7,8^. PSA level also tends to rise in men with benign prostatic hyperplasia (BPH) and as such it is a good marker for prostate volume and also in acute bacterial prostatitis. Recently, other factors that influence the ratio free PSA/total PSA in serum are age, prostate size, drug treatment, prostate manipulation, sample stability or assay determination method^9^. As PSA effectiveness in the identification of the malignancy is diminished by its low diagnostic specificity, free PSA measurements in serum have been proposed for improving the specificity of laboratory identification of prostate cancer. The percentage of measured prostate-specific antigen (free/total PSA ratio) is useful in assessing the risk of prostate cancer in patients with borderline or moderately increased total PSA (4.0-10.0 ng/mL) and has been used to help select men who should have follow-up prostate biopsy^10^. Higher total PSA levels and lower percentages of free PSA are associated with higher risks of prostate cancer^11,12^.

## MATERIALS AND METHODS

### Study population

This retrospective study was conducted in collaboration with urology unit in Durres Hospital in Albania. All patients included in this study were admitted from January 2018 to December 2020. The study was based on data collected from 1201 male patients admitted at outpatient clinic, between 35 and 84 years of age (mean age 63 years) with suspect or documented medical history regarding prostate evaluations. The inclusion criteria were based on the availability of complete clinical records and the patient’s consent to undergo further testing for new markers of prostate cancer. Patients presented with concomitant disease or evidence of prostate manipulation especially needle biopsy and transurethral resection (TRU) two weeks prior to blood collection were not included in the study. Also, men treated for benign prostatic hyperplasia with inhibitors of 5-alpha-reductase (finasteride) and patients receiving treatment with androgens and luteinizing hormone-releasing factor agonists were excluded from this study.

### Study group

This study involved 1038 patients who underwent laboratory examinations to determine Total PSA concentrations and, in some cases, free PSA determinations. Furthermore, this study involved an additional 163 patients who had undergone only free PSA measurements. Further subgrouping of patients was performed on the basis of age and availability of data regarding measurement of free PSA.The first subgroup involved 511/1038 patients who were further stratified according to two different age groups and who had determinations of Total PSA concentrations. The second subgroup included 69/1038 patients who had both, Total and Free PSA determinations. Further analysis was done by evaluating data of this subgroup according to age. On the other hand, separate statistical analysis was performed on the 163 patients who had only free PSA concentrations determined.

### Sample collection, processing, and storage

Venous blood samples were collected following an overnight fasting using standardized procedure forcollection and storing blood samples.

### PSA and Free PSA analysis

Total PSA and Free PSA serum concentrations were measured using Roche Cobas e-601 instrument. The Roche Elecsys Total PSA (prostate-specific antigen) method is a sandwich electrochemiluminescence immunoassay that employs a biotinylated monoclonal PSA-specific antibody and a monoclonal PSA-specific antibody labeled with ruthenium complex. A direct relationship exists between the amount of Total PSA and Free PSA respectively antigen in the sample and relative light units. This method has been standardized against the Reference standard WHO 96/670. (Package insert: Elecsys total PSA reagent, Roche Diagnostics, Indianapolis, IN, V 1.0, 07/2018).

### Statistical Analysis

Statistical Minitab 17 Statistical Software Version was used for the provide statistical analysis of data which included descriptive statistics (frequency, mean, and standard deviation). Analysis of variances was used to gain information about the relationship between variables. The degree of correlation between two variables was determined with the use of Pearson's correlation. Statistical significance was considered if p< 0.05.

## RESULTS AND DISCUSSION

### Total PSA levels in all patients (N=1038)

**Figure 1:**
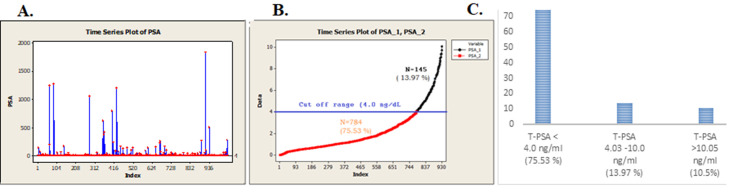
Distributions profile for all Total PSA values according to cut-off and selected concentration intervals A. Distribution profile of Total PSA concentrations in 1038 serum samples; B. Distribution profile of Total PSA concentrations with values falling between 0.03-10.00 ng/m; C. Total PSA values expressed as percentages according to reference intervals.

The Total PSA concentrations of all 1038 patients according to cut-off and selected concentration intervals are shown in Figure 1. Based on manufacturer’s instructions, the concentrations of 4.0 ng/mL and 0.5 ng/mL were considered as the upper limits (cut-off values) of normality for Total PSA and Free PSA, respectively^13^.According to our data, 75.62% of patients (785/1038) had Total PSA concentrations within the cut-off value of 4.0 ng/ml. Whereas the other 24.37% (253/1038) had Total PSA concentrations exceeding the cut–off value (Figure 1). Total PSA values between within the interval 4.0-10.0 ng/ml have been of interest during this study, due to the fact of being described as the diagnostic "gray zone," by urologists. In these diagnostic interval Free/Total PSA ratio helps determine the relative risk of prostate cancer^14^. In addition, the distributions of all total PSA values less than 10 ng/ml are represented also in Figure 1B. As shown by the distribution of values ​​defined by the range 0.03 -10.00 ng/mL, there are a total of 929 patients whose Total PSA values are within the specified limits. Concentration of < 4 ng/ml was determined in 75.53 % of the patients (N_<4 ng/mL_ = 784). Whereas 13.97% of patient (N_<4 -10ng/mL_ = 145) had concentrations within the 4 - 10 ng/ml. Patients with Total PSA values higher than 10 ng/ml were only 10.50% out of the total 929 individuals (N >10.0 ng/ml =109) indicating higher probability of cancer which would requires further prostate evaluations^15^. All data are shown as percentages in Figure 1.In order to obtain more information regarding the distribution of Total PSA values all data were evaluated using Descriptive Statistics and results are presented in Table 1. As shown from our data, Total PSA concentrations had substantial variability (CV=640%) marked by large differences between PSA values as represented by the extensive interval (min 0.03 ng/ml and max 1835 ng/mL). As such it can be concluded that Total PSA values among 1038 patients are characterized by an unsymmetrical distribution as defined by the calculated statistical parameters such as skewness 11.87 (>2) and Kurtosis 160.88(>3).Descriptive statistics shown in Table 2 refer to data related to Total PSA concentrations measured as according to the specified range interval (0.03-4.0 ng/mL, 4.05-10.00 ng/mL and > 10.0 ng/mL).According statistical data represented in Table 2. mean Total PSA values were 1.42 ng/mL and 6.06 ng/mL for measuring intervals 0.03-4.00 and 4.05-10.00 ng/ml respectively. The concentration of Total PSA, combined with patient age, family history, ethnicity, and other factors, can be used to determine subsequent follow-up^16^. Also, Total PSA values found within 0.03-4.00ng/ml interval are characterized by a normal distribution as indicated by Skewness values < 2, and Kurtosis value of < 3. Further analysis showed moderate variability, CV=68.10 % and 93.13% for intervals 0.03-4.0 ng/ml and 0.03-10.0 ng/ml respectively. These findings may be justified by the slightly lower average age of patients included in the groups categorized by interval ranges of younger patients and the existing guidelines which indicate lower chances for prostate cancer in patients with Total PSA values less than 2.0 ng/ml^17^.

**Figure 2:**
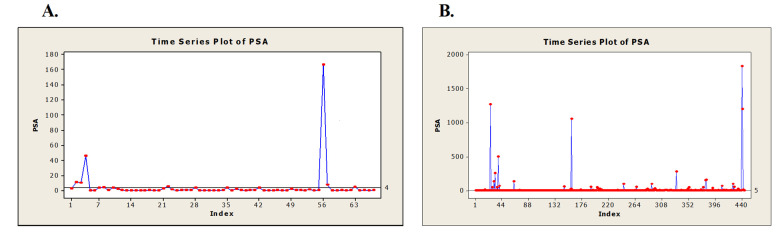
Distribution profile of Total PSA concentrations in patient according to age compare with cut-off value **A**. Distribution profile of Total PSA concentrations in patients under 50 years old compared with cut-off values (4.0 ng/mL); **B**. Distribution profile of Total PSA concentrations in patient over 50 years old compared with cut-off values (4.0 ng/ml).

**Total PSA values according to age**

**Table 1:** Descriptive Statistics for Total PSA values of all patients included in the study

Variable	Total count	Mean	Median	StD	Variance	CV %	Min	Max	Skewness	Kurtosis
Total PSA	1038	16.17	1.63	103.63	1038.99	640.95	0.03	1835	11.87	160.88

**Table 2:** Descriptive Statistics for Total PSA values according to selected concentration intervals

Variable Total PSA	Total count	Mean	Median	StD	Variance	CV %	Min	Max	Skewness	Kurtosis
0.01-10.00 ng/ml	929	2.16	1.41	2.00	4.030	93.13	0.00	10.05	1.59	2.15
0.01-4.00 ng/ml	784	1.42	1.16	0.97	0.9241	68.10	0.00	3.96	0.79	-0.26
4.05-10.05 ng/ml	145	6.06	5.72	1.56	2.45	25.74	4.05	10.00	0.71	-0.45
>10.05 ng/ml	109	136.9	34.03	296.0	87621.5	216.22	10.03	1835	3.71	14.64

**Table 3:** Descriptive Statistics for Total PSA values according to age

Variable Total PSA	Total count	Mean	Median	StD	Variance	CV %	Min	Max	Skewness	Kurtosis
Patients under 50 years old	67	4.96	0.93	20.95	438.71	93.13	0.26	167	7.35	56.56
Patients over 50 years old	444	21.08	1.77	133.7	17891.2	634.54	0.03	1835	10.26	114.37

The concentrations of Total PSA were further analyzed according to age. As mentioned above, 511/1038 patients were separated into two groups, one being less than 50 and the other one above 50 years of age. as affected by age. Descriptive statistics was used to evaluate the distribution of data according to age as shown in Table 3 and Figure 2.Based on data presented in Table 3, there is a substantial difference in the mean Total PSA values between patients whose age was less than 50 and those who were above 50 years of age. The mean T-PSA values in these two groups were 4.96 ng/ml and 21.08 ng/ml respectively, whereas the median was 0.96 ng/ml and 1.77 ng/ml. These findings are in concordance with other authors in which the normal interval reference for patients less than 50 is < 4.0 ng/ml with a median of 0.9 ng/ml^18^. Age-specific PSA reference ranges are a result of the increasing mean PSA and increasing PSA variance in successively older cohorts of men^19^. Referring to data collected it was observed a high variance and standard deviation of 20.95 ng/ml for patients under 50 years and a standard deviation of 133.76 ng/ml in patients over 50 years of age. The variability in PSA values in males as their age progresses suggests that caution should be exercised when considering the clinical implications of age-specific reference ranges^20^.

### Free PSA tumor marker test

**Figure 3:**
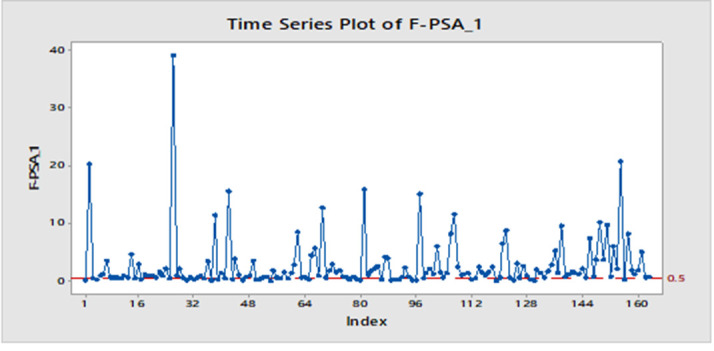
Distribution profile of Free PSA concentrations in all serum samples compared with cut-off values (0.5 ng/mL)

Free PSA measurements in serum have been performed to improve the specificity of laboratory examination which support the clinical findings towards identification of prostate cancerin patients with Total PSA ≥ 4.0 ng/mL**. **Hence, data from 163 patients who underwent only free PSA determinations were analyzed statistically to evaluate the distribution and specific characteristics of data as shown in Figure 3. and Table 4.As shown data presented above, there is a substantial variability among observed results of free PSA which further indicates unsymmetrical distribution of data. The mean free PSA value was 2.67 ng/ml and the range interval was limited by 0.003 and 39.05 ng/ml.

**Table 4:** Descriptive Statistics for Total PSA values according to age

Variable	Total count	Mean	Median	StD	Variance	CV %	Min	Max	Skewness	Kurtosis
F-PSA ng/ml	163	2.67	1.07	4.66	21.70	174.53	0.003	39.05	4.28	25.08

**Table 5:** Descriptive Statistics for Total PSA values according to selected concentration intervals

Variable	Total count	Mean	Median	StD	Variance	CV %	Min	Max	Skewness	Kurtosis
Total PSA Ng/ml	69	55.2	3.7	143.6	40628.3	260.32	0.03	654	308	8.74
F-PSA ng/ml	69	3.071	0.803	6.937	48.116	225.88	0.01	39.05	3.93	16.49
T-PSA/F- PSA ratio %	69	22.50	22.05	12.08	145.99	53.71	0.25	63.20	0.55	1.31

### Total and free PSA determinations among 69 patients (second subgroup)

In the second subgroup, involving 69 out of 1038 patients’ further analysis was performed and reflected in the Table 5. As stated above, all 69 patients had both measurements of free PSA and Total PSA determined. Also, it worth noting that all 69 patients had Total PSA values within the measuring interval of 4.0-10.0 ng/mL/. Hence the ration Free/Total PSA was determined as to further evaluate the risk for prostate cancer. As shown from statistical data extrapolation the percent of free PSA/total PSA ratio lower than 10% is observed only in 14.49 % (10/69) patients whereas the other 85.50% (59/69) patients had percent free PSA higher than >10% despite the fact that Total PSA values of such patients were within 3.0-10.0 ng/ml interval. The data are represented inFigure 4.In agreement with our finding and as stated by other authors all patients whose Total PSA values fall within 3.0-10.0 ng/ml, and percent free PSA in normal range are advised to have regular annual evaluation to determine the Total PSA changes as time progresses. In contrary patients with percent free PSA <19%, and Total PSA value between 4-10 ng/ml need to undergo DRE (digital rectal examination) and TRUS (transrectal ultrasonographic)^18^.As reflected by data presented in Table 5, Free and Total PSA values among 69 patients have an abnormal distribution of total PSA and Free PSA values measured, which may relate to various factors that influence these markers^13^. On the other hand ratio of Free PSA/Total PSA shows a normal distributions as indicated by skewness < 2.0 and kurtosis <3.0. The distribution histograms of Free, Total PSA and their ratio is shown in Figure 5. Distribution histograms of F-PSA/T-PSA ratio reflects that data are centered around 10 to 20 % value indicating a normal distribution pattern falling withing the range of 0.25 to 63.20%. Total PSA and Free PSA values were positively skewed on the left (righ skewed) with a high variance and a high variability (CV 260.32 %) and (225.88 %) of both Total-PSA and Free-PSA respectively, indicating once again the abnormal distribution of data in our study. These findings are in compliance with other studies which emphasize that high total PSA levels associate with low free PSA levels, thus generally indicating a risk for prostate cancer. As claimed by other authors the usefulness of percent free PSA to improve cancer detection when the total PSA value is within normal range is evidence-based^21^. Data showing high variability among Total and Free PSA values are shown through the distribution probability graphs and boxplots in Figure 6.According to data shown in Figure 6 parallel determinations of total and free PSA values reduces further follow up procedures in 24.599 % of patients with Total PSA levels more than 4.03 (56.4891%) and F-PSA levels less than 0.52 ng/ml (31.89 %). Therefore, it can be concluded that F-PSA is a good marker for stratifying patients with prostate cancer risk. These findings are in compliance with other authors who have concluded that the risk of having prostate cancer may be as high 56 percent when the ratio of free to total PSA is between 0 and 10 percent, in addition, in cases with ratio greater than 25 percent, this risk reduces to 8 percent^13,21^. As show in box plots graphs (Figure 6B) T-PSA and F- PSA values have medians of 3.89 and 0.803 respectively. Furthermore, medians for F- PSA/ T-PSA ratio was 22.30. For three parameters the plot displays outliers. In agreement with our findings other authors have shown the usefulness of percent free PSA in reducing unnecessary biopsies in patients undergoing evaluation for prostate cancer^14,22^.

**Figure 4:**
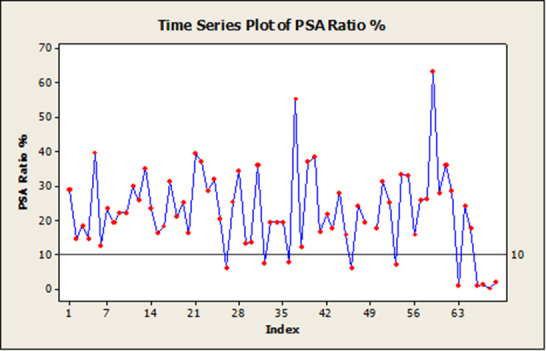
Distribution profile of Free PSA concentrations in all serum samples compared with cut-off values (0.5 ng/mL)

**Figure 5:**
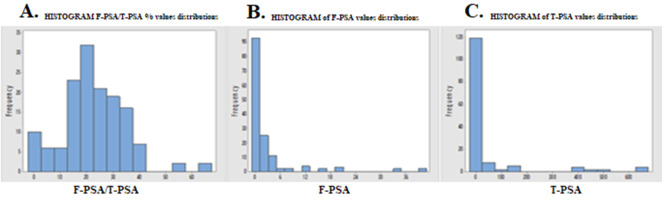
Histogram of distributions for all study parameters in 69 patients **A.** Histogram of distributions for F-PSA/T-PSA ratio; **B.** Historam of Distributions for F-PSA; **C.** Histogram of distributions for T-PSA values in 69 patients

**Figure 6:**
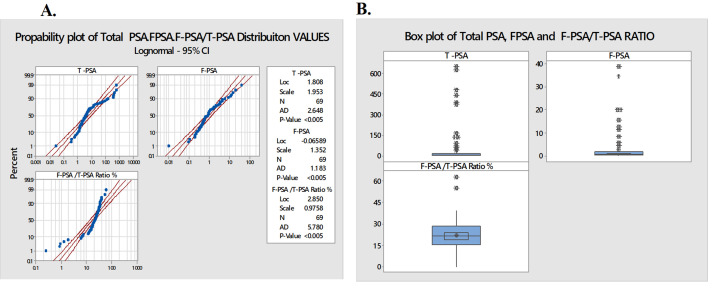
Distribution probability of values measured for all study parameters in 69 patients. **A. **Distribution probability graphs for T-PSA, F-PSA, F-PSA/T-PSA ratio log normal-95% CI; **B.** Distributions box plots graphs for T-PSA, F-PSA, F-SA/T-PSA ratio in 69 patients.

**Figure 7:**
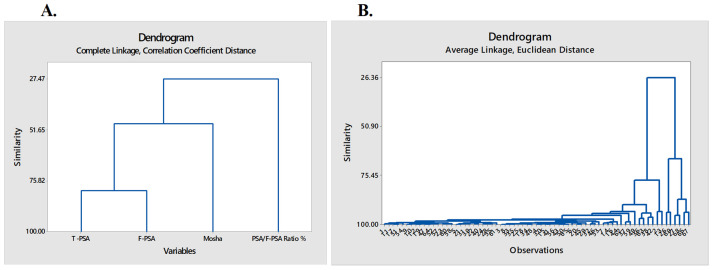
Cluster Analyses of study parameters **A**. Dendogram complete linkage correlation coefficient distance for T-PSA; F-PSA/T-PSA; AGE, parameters; **B.** Dendogram Average linkage Euclidian distance between observations.

**Table 6:** Correlation analysis between T-PSA and F-PSA: T-PSA and F-PSA/T-PSA ratio regardless age

Variables	Sample size	Correlation coefficient r	Significance level	95% cofidence interval for r
T-PSA and F-PSA regardless age	69	0.7938	P< 0.0001	0.6860 to-0.8675
T-PSA and F-PSA/T-PSA ratio regardless age	69	-0.6275	P<0.0001	-0.7525 -0.4590

**Figure 8:**
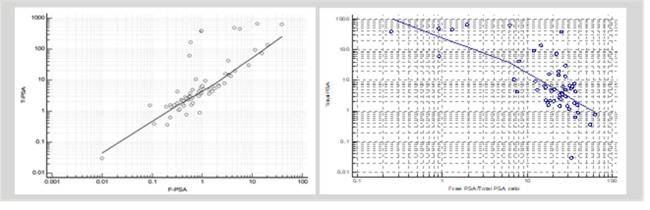
Correlation analysis between parameters regardless age A. Correlation analysis between T-PSA and F-PSA regardless age B. Correlation analysis between T-PSA and F-PSA/T-PSA

### Multivariable analysis and data correlation.

From a different approach, analysis based on statistical processing in clusters provides detailed information regarding the similarity of Free and Total PSA measurements which are shown to have a similarity level of 80.42% which is above the criteria for classification of parameters in groups, according to similarity level. The high similarity between Total PSA and Free PSA leads to the assumption that these two parameters depend on common factors. The two other variables age and Free PSA / Total PSA ratio are grouped in separated cluster (Figure 7).

**Table 7:** Correlation analysis between T-PSA and F-PSA and F-PSA/T-PSA ratio according to age

Variable	Sample size	Correlation coefficient r	Significance level	95% cofidence interval for r
Total PSA and F-PSA/ T-PSA ratio in patients over 50 years old	53	-0.6969	P< 0.0001	-0.8139 to 0.5256
T-PSA and F-PSA/ T-PSA ratio among patients ≤ 50 years old	16	0.2826	P=0.2890	-0.2479 to 0.6827

**Figure 9:**
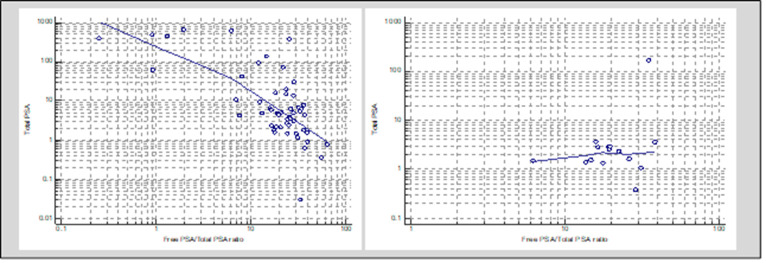
Correlation analysis between parameters according to age **A.** Correlation analysis between Total PSA and F-PSA/T-PSA ratio in patients over 50 years old 53/69; **B.** Correlation analysis between T-PSA and F-PSA/T-PSA ratio among patients ≤ 50 years 16/69.

### Correlation analysis of Total and Free PSA

Furthermore, a correlation analysis was performed between Total PSA values and Free PSA/Total PSA ratio regardless of age and according to age. Data from correlation analysis are presented in Table 6 and Figure 8. The relationship between T-PSA, F-PSA ratio and age was evaluated statistically based on data from other studies indicating that Total PSA values are age depended^23^. Further studies have shown that even though percent free PSA and Total PSA vary with age Free/Total ratio remains an independent variable^11^. Data generated from samples and stratified by age are represented in Table 7and Figure 8.As shown in the correlation analysis there is a statistically significant (p< 0.0001), correlation between T-PSA and F-PSA (r= 0.7983) and meanwhile a statistically significant (p< 0.0001) but negative correlation (r= - 0.6275) was found between Total PSA and Total PSA/Free PSA ratio patients who had both Total and Free PSA examinations performed. When stratifying by age, this correlation becomes stronger (r= - 0.6969 and p< 0.0001) for patients over 50 years of age. On the contrary, the correlation becomes weak and statistically insignificant (r= 0.2826; p= 0.2890) among patients underage of 50.

## CONCLUSION

In 75.62 % of patients (785/1038 cases) Total PSA concentration did not exceed the cut-off value. 4.0 ng/mL In 24.37 % of patients (253/1038 cases) Total PSA concentration exceeded the cut–off valueTotal PSA values during this study have an unsymmetrical distribution. In addition, patients who values of Total PSA were < 4 ng/mL comprise 84.4 % (785/1038) of total cases. Patients with values <10 ng/ml were 89.69% (931/1038). Furthermore, there were 145 patients (15.6%) whose Total PSA values were between 4-10 ng/ml. Lastly, only 10.32% (107/1038) of patients had values of Total PSA > 10 ng/ml which may be indicative of cancer and as such require examinations including prostate biopsy. Measuring serum free PSA concentrations along with PSA concentrations may provide higher accuracy for detecting prostate cancer and might eliminate unnecessary biopsies in men with Total PSA level of more than 4.0 ng/ml. Several studies showed that the percentage of free to total PSA was lower in cancer than in non-cancer cases^22^. Our study is in agreement with such findings. Further studies have demonstrated the utility of percent free PSA in reducing unnecessary prostate biopsies. Percent free PSA values varying from 14% to 28% were shown to, avoid 20% to 65% of unnecessary prostate biopsies^24^. On the other hand, our study, which evaluated cases with percent free PSA value of 10% in patients with Total PSA >4.0 ng/ml showed to reduce the unnecessary prostate biopsies by 85.5%. Similar to other studies, our data suggested that percent of F-PSA improves diagnostic effectiveness of T-PSA in patient with low T-PSA and that there is a need to optimise the diagnostic performance of percent F-PSA assay by controlling methodological variability by avoiding the impact of preanalitical and analytical variables reviewed recently^25^.

## Recommendations and limitation

With Prostate cancer disease being one of the major causes of death among men worldwide, there is an ongoing need for new biomarkers that are able to assist in clinical decision making. A major focus in urologic research is the identification of new biomarkers with improved specificity for clinically significant prostate cancer^25,26^. A promising new test based on prostate-specific antigen (PSA) is called the Prostate Health Index (PHI), which has recently been approved in the United States, Europe and Australia. PHI is a mathematical formula that combines total PSA, free PSA and [-2] proPSA. This new blood test represents significant promise for both prostate cancer screening and treatment decision-making^27^. The addition of this parameter would further complement the findings reported in our study.Our study would provide more comprehensive data if interval specific blood drawing had to be implemented. This would provide further insight on how the tumor markers evaluated in our study would fluctuate with time.

## KEY POINTS

**◊** *Measuring serum free PSA concentrations along with PSA concentrations may provide higher accuracy for detecting prostate cancer and might eliminate unnecessary biopsies in men with Total PSA level of more than 4.0 ng/mL.*

**◊** *Our data regarding cases with percent free PSA value of 10% in patients with Total PSA >4.0 ng/ml showed to reduce the unnecessary prostate biopsies by 85.5%.*

**◊** *Our study suggests that percent F-PSA improves diagnostic effectiveness of T-PSA in patient with low T-PSA and that there is a need to optimize the diagnostic performance of percent F-PSA assay by reducing the impact of preanalytical and analytical variables.*
